# Combination of compressed sensing and parallel imaging with respiratory motion correction for highly-accelerated cardiac perfusion MRI

**DOI:** 10.1186/1532-429X-13-S1-O98

**Published:** 2011-02-02

**Authors:** Ricardo Otazo, Daniel Kim, Leon Axel, Daniel K Sodickson

**Affiliations:** 1 New York University School of Medicine, New York, NY, USA

## Introduction

Cardiac perfusion MRI requires fast data acquisition to achieve an appropriate combination of temporal resolution, spatial resolution and spatial coverage for clinical studies [1]. We have recently presented a combination of compressed sensing and parallel imaging (k-t SPARSE-SENSE) to highly accelerate perfusion studies [2]. However, this method is sensitive to respiratory motion, which decreases temporal sparsity and produces temporal blurring in the reconstructed images. In this work, we present a rigid respiratory motion correction method which allows highly-accelerated first-pass cardiac perfusion MRI to be performed without strict breath-holding.

## Purpose

To develop a respiratory motion correction method for joint compressed sensing and parallel imaging acceleration of first-pass cardiac perfusion MRI.

## Methods

Free-breathing first-pass cardiac perfusion MRI with 0.1 mmol/kg of Gd-DTPA (Magnevist) was performed using a modified multi-slice TurboFLASH pulse sequence. Healthy volunteers were imaged on a whole-body 3T scanner (Siemens; Tim-Trio) using the standard 12-element body matrix coil array. The relevant imaging parameters include: FOV = 320mmx320mm, image resolution = 1.7mmx1.7mm, slice-thickness = 8mm, TE/TR = 1.3/2.5ms, repetitions=40. An acceleration factor of 8 was used to acquire 10 slices per heartbeat with temporal resolution of 60ms/slice. Data undersampling was performed using a pseudo-random ky-t pattern [2]. Fully-sampled low-resolution coil sensitivity reference data were acquired in the first heartbeat. Image reconstruction was performed in two-steps using the k-t SPARSE-SENSE algorithm [2] with temporal FFT as sparsifying transform. First, an intermediate k-t SPARSE-SENSE reconstruction is generated for respiratory motion correction. Rigid motion between frames is detected by computing the displacement of each frame from this intermediate k-t SPARSE-SENSE reconstruction with respect to the coil sensitivity reference using a crosscorrelation approach in the image domain [3]. Second, motion correction is performed by aligning all the frames in the accelerated data. The final k-t SPARSE-SENSE reconstruction is computed using the aligned accelerated data.

## Results

**Figure 1:**
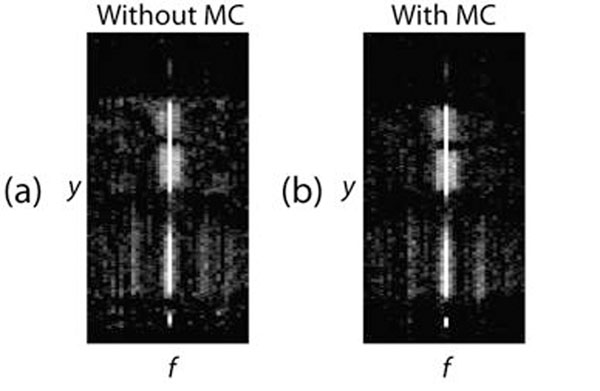
Sparse y-f domain for intermediate k-t SPARSE-SENSE reconstruction with (a) no motion correct and (b) motion correction.

**Figure 2:**
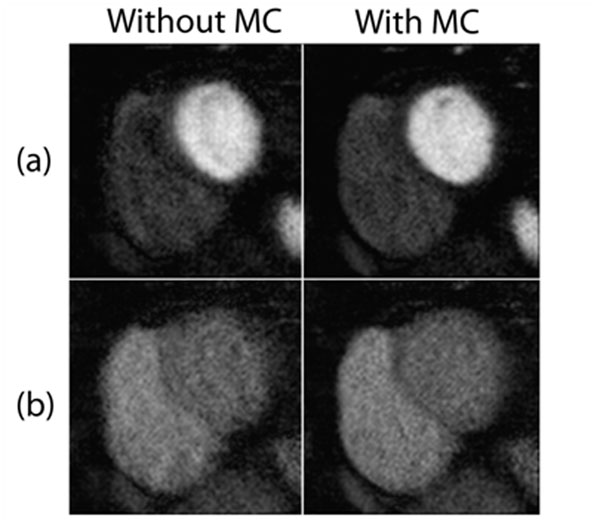
k-t SPARSE-SENSE reconstruction of a representative slice without and with motion correction (MC) for (a) peak blood enhancement and (b) myocardial wall enhancement.

Rigid respiratory motion correction significantly increased sparsity in the temporal Fourier domain, which is due to better alignment among frames (Fig. 1). Fig. 2 shows k-t SPARSE-SENSE reconstruction of a representative slice from the free-breathing perfusion scan without and with motion correction. The utilization of motion correction decreased temporal blurring and presented images with higher quality.

## Conclusions

This work demonstrates feasibility of highly-accelerated first-pass cardiac perfusion MRI without strict breath-holding with rigid respiratory motion correction. Future work will explore the use of non-rigid motion correction. The proposed technique may be useful for imaging patients with impaired breath-hold capabilities.
